# Tips and Pitfalls of Surgical Techniques for Scoliotic Deformities in Neurofibromatosis Type 1

**DOI:** 10.3390/jcm15010104

**Published:** 2025-12-23

**Authors:** Angelos Kaspiris, Ioanna Lianou, Vasileios Marouglianis, Roberta-Spyridoula Afrati, Evangelos Sakellariou, Andreas Morakis, Panagiotis Karampinas, Elias S. Vasilisadis, Spiros G. Pneumaticos

**Affiliations:** 1 Third Department of Orthopaedics and Medical School, National and Kapodistrian University of Athens, KAT General Hospital of Athens, Nikis 2, 14561 Athens, Greeceandreasmorakis@gmail.com (A.M.); karapana@med.uoa.gr (P.K.);; 2 Department of Orthopaedics, “Agios Andreas” General Hospital of Patras-NHS, 262224 Patras, Greece; jlianou@med.uoa.gr; 3 Postgraduate Programme for “Rehabilitation Following Spinal Cord Lesions, Spinal Pain Management”, Third Department of Orthopaedics and Medical School, National and Kapodistrian University of Athens, KAT General Hospital of Athens, Nikis 2, 14561 Athens, Greece

**Keywords:** neurofibromatosis type 1, scoliosis, dystrophic, curves, Cobb angle

## Abstract

**Background**: Neurofibromatosis 1 is an autosomal dominant disorder accompanied by extensive early-onset spinal manifestations, with or without dystrophic scoliotic features. While non-dystrophic subtypes can often be treated similarly to idiopathic scoliosis, dystrophic scoliosis typically requires more aggressive intervention, often involving instrumentation in severely compromised pedicles or vertebrae. **Purpose**: This review aims to present recent advances in the surgical treatment of Neurofibromatosis 1-associated scoliosis, including surgical techniques and emerging guidance methods. **Methods**: An electronic literature search was conducted in Web of Science and PubMed to identify surgical techniques for scoliosis in patients with Neurofibromatosis 1. **Results**: Forty-one studies on the operative treatment of dystrophic scoliosis or both subtypes were retrieved. Although aggressive treatment with combined anterior and posterior fusion are widely used, posterior-only methods, which avoid plexiform tumours, present encouraging results. Recent studies highlight the effectiveness of growing rod systems in early-onset cases, enabling delayed fusion while preserving T1-S1 growth. Promising results from sectional or segmented correction techniques demonstrate better sagittal balance and Cobb angle correction, respectively. Preoperative use of halo-gravity traction, which has been extensively studied, is associated with reduced neurological impairment and encourages better correction results, avoiding autofusion. Various studies have also reported more precise pedicle screw placement with guidance of O-arm and triggered electromyography (t-EMG). **Conclusions**: The correction of spinal scoliotic deformities presents a significant challenge. However, recent advances in surgical techniques and intraoperative guidance offer promising strategies for more effective management.

## 1. Introduction

**Figure 1:**
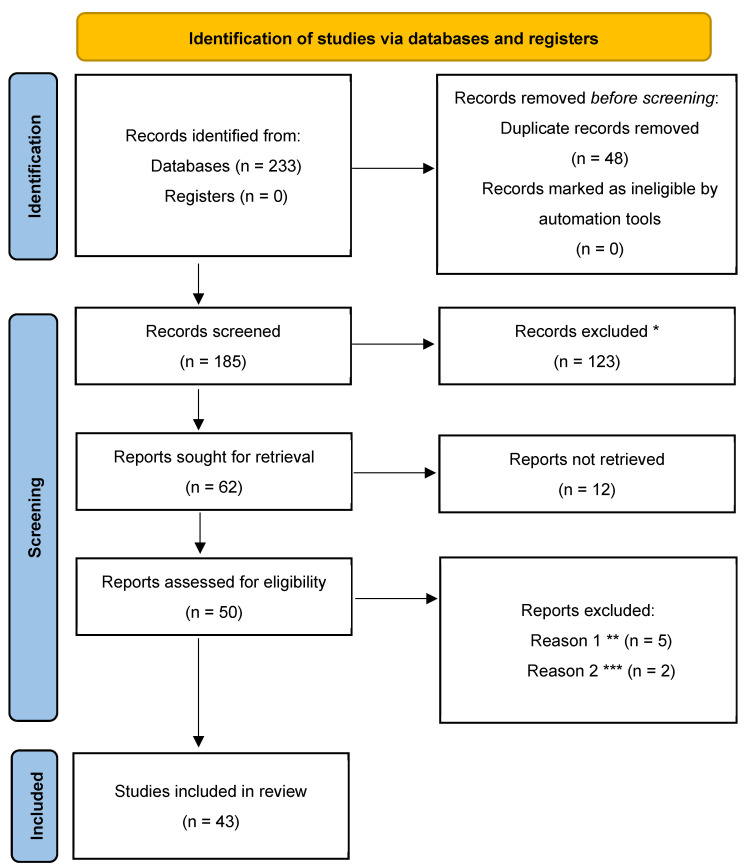
PRISMA 2020 flow diagram illustrating the methodology of the systematic review on surgical treatment strategies for scoliosis associated with neurofibromatosis type 1. The diagram includes searches of databases and registers only. (* Records excluded based on title/abstract screening, ** Records referring to spinal deformities other than scoliosis *** Records excluded, due to wrong population setting).

**Figure 2:**
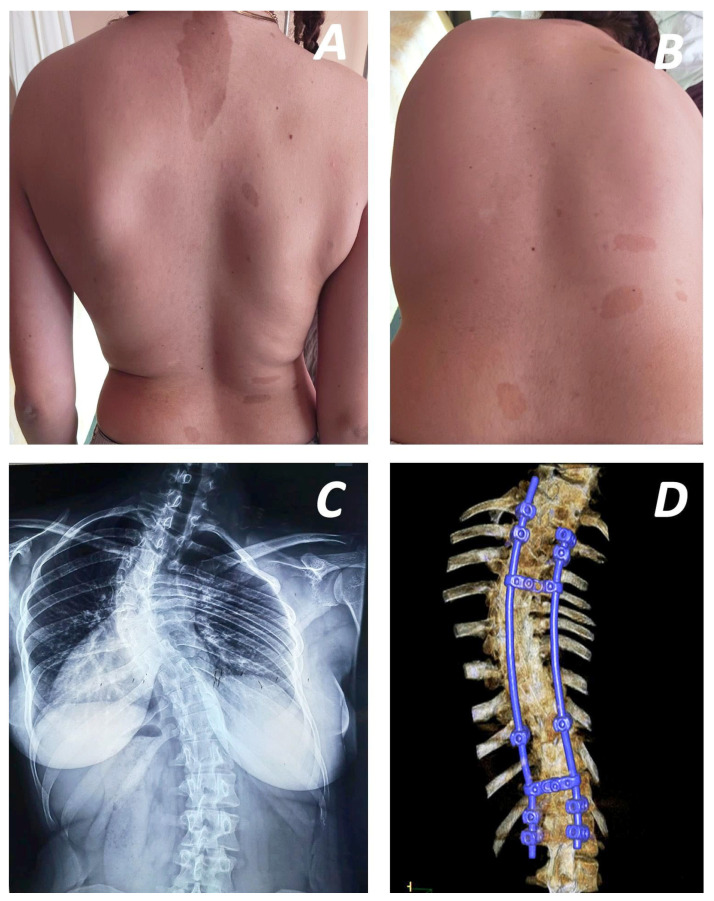
27-year-old patient suffering from dystrophic scoliotic deformity without neurological symptoms with the characteristic flat, café-au-lait spots and freckles on the skin, presenting left shoulder elevation (**A**) and hub at the left side of the back during Adam’s forward bend test (**B**). The radiographic examination revealed left thoracic scoliotic deformity of 76° between vertebrae T3 and T10 with the apex at T6 (**C**). Surgical correction with posterior approach and instrumentation between T1 and L2 led to partial reduction in scoliosis of 42° due to limited bony stock and hypoplastic pedicles (**D**).

Neurofibromatosis is classified into two subtypes: Neurofibromatosis type 1 (NF1) or peripheral subtype, which affects the vestibular nerve, and Neurofibromatosis type 2 (NF 2), known as central neurofibromatosis [1]. NF1, first described in 1882 by von Recklinghausen, is an autosomal dominant disorder, caused by mutations in the NF1 gene located on chromosome 17q11.2 [2]. This mutation causes loss of function of the tumour suppressor gene NF1, which is expressed in a wide variety of cells. The NF1 gene encodes neurofibromin, a cytoplasmic protein that downregulates cellular proliferation, differentiation, and growth through inactivation of the Ras-GTPase protein and accumulation of cyclic adenosine monophosphate (cAMP). Aberrant activation of the Ras pathway leads to upregulation of Ras-Raf-MEK-ERK and cross-talk with the phosphatidylinositol-3-kinase (PI3K) pathway [3,4]. Given the extensive expression of NF1 in various cell types, the disease exhibits heterogeneous penetrance and unpredictable manifestations (Figure 1) in different systems [5]. Its estimated incidence is approximately 1 in 2500 to 3000 individuals, who typically present with a variety of clinical manifestations including nerve-related complications such as non-malignant cutaneous or plexiform neurofibromas, freckles and café-au-lait spots (Figure 2A), optic gliomas, various skeletal disorders, most notably, spinal deformities (Figure 2B), and even malignant peripheral nerve sheath tumours in some cases [2,6].

Spinal deformity is the most common osseous manifestation of NF1, occurring in approximately 10–60% of affected individuals [7]. NF1-related scoliosis (Figure 2C) accounts for approximately 3% of all scoliosis cases [8]. The deformity is typically classified as either dystrophic or non-dystrophic, based on the presence of characteristic osseous dystrophic features. Dystrophic scoliosis is defined by the presence of vertebral dysplasia, commonly presenting with dural ectasia, paravertebral neurofibromas, vertebral scalloping, and rib pencilling, among other features. Non-dystrophic curves generally follow a more benign course and share similarities with idiopathic scoliosis. In contrast, dystrophic scoliosis presents at an earlier age and tends to be more aggressive. This type of scoliosis consists of a short-segmented curvature with sharp angulation and wedged vertebrae, intraspinal or paraspinal soft tissue masses accompanied by severe rotation of the vertebral column and an enlarged intervertebral foramen [9]. Additional findings may include rib dislocation, the “pencil sign”, and, in rare cases, rib head protrusion into the spinal canal—a complication documented in only 22 cases in the literature [1]. 

The presence of intraspinal ribs can be identified through MRI (Magnetic Resonance Imaging) and CT (Computed Tomography) scanning, although most patients are asymptomatic. However, cases of spinal cord compression with concomitant hemiplegia and paraplegia have been reported in the context of pre- or postoperative complications [10]. Conservative treatment with bracing is typically ineffective, and the severity of these deformities often necessitates early and usually aggressive surgical intervention [6]. Treatment strategies have traditionally focused on spinal fusion interventions with pedicle screw or hook instrumentation via posterior or anterior approaches. However, early-onset spinal deformities necessitate growth-friendly procedures, such as the use of growing rods, which allow more normal truncal and chest growth [11]. 

The aim of this review is to present the most recent updates on tips and pitfalls in the surgical management of scoliotic deformities associated with NF1, with a focus on both dystrophic and non-dystrophic subtypes. A range of instrumentation methods, surgical approaches, preoperative planning tools and intraoperative guidance systems are analysed, reflecting the complexity inherent in surgical management for patients with NF1. To our knowledge, this the first comprehensive literature review addressing surgical techniques and instrumentation employed in the treatment of NF1-related scoliosis.

## 2. Materials and Methods

### 2.1. Systematic Literature Review

A computer-based literature review was conducted on 10 July 2025 in accordance with the latest PRISMA (Preferred Reporting Items for Systematic Reviews Meta-Analyses) guidelines (Supplementary Materials). The results were presented in a PRISMA flow chart (Figure 1). The databases searched were PubMed (1947 to present) and Web of Science (1900 to present). All studies were retrieved following a comprehensive search methodology using the combination of the terms and phrases “neurofibromatosis type 1 [All fields]”, “surgery [All fields]”, “scoliosis [All fields]” and “spinal deformities [All fields]”. 

The review protocol was registered in the International Prospective Register of Systematic Reviews (PROSPERO) under registration number CRD420251107808 with full public access available.

### 2.2. Inclusion and Exclusion Criteria

Inclusion criteria were as follows: (a) full texts only, (b) original articles, (c) case reports or case series, (d) comparative studies regarding the use of different methods of treatment of both dystrophic and non-dystrophic subtypes of NF1-associated scoliosis, (e) studies focusing on all instrumentation methods, surgical approaches, treatment options (fusion procedure or growth-friendly methods), preoperative planning or intraoperative imaging tools. No restrictions were applied regarding patient age or publication date. 

Exclusion criteria were as follows: (a) laboratory studies or studies based on in vitro or in vivo models, (b) letters to the editor, editorials, technical notes, book chapters, and expert opinions, (c) studies without sufficient data regarding the exact type of spinal deformity in patients with confirmed diagnosis of NF1, with insufficient data regarding the neurofibromatosis subtype and the exact type of surgical intervention, (d) studies incorporating population with kyphotic, or population with mixed features; kyphosis and kyphoscoliosis, (e) studies in languages other than English. 

According to Lykissas et al. and Duranni et al., scoliotic deformity is characterised as dystrophic when three of the following criteria are present: (a) depth of vertebral scalloping greater than 3 and 4 mm in the thoracic and lumbar spine, respectively, (b) pencilling of rib, (c) transverse processes spindling, (d) rotation of vertebrae (grade 3 or more, according to the Moe–Nash method), (e) short segmented curvature (six or fewer vertebrae), (f) paraspinal tumours or plexiform neurofibromas, (g) dural ectasia, (h) wedged vertebrae, (i) dysplasia of the pedicles, (j) widening of the interpediculate distances, and (k) widening of the intervertebral foramina [12,13]. All studies were further categorised accordingly and the subtypes of the deformity included was stated (Table 1).

### 2.3. Data Extraction and Quality Assessment

The literature search and data extraction were independently performed by two reviewers and a librarian. Based on the inclusion and exclusion criteria, titles and abstracts from relevant articles were screened and eligible data were recorded in a Microsoft Excel spreadsheet (Microsoft Office 365, Redmond, WA, USA). Disagreements regarding study inclusion or data extraction were resolved by the senior author. This study was conducted according to the principles of systematic reviews. 

### 2.4. Evaluation of Study Quality

The methodology of the selected studies was independently evaluated using the Newcastle–Ottawa quality assessment scale. With the exception of review articles, studies were categorised into three quality levels: scores of 0–3 were classified as poor, scores of 4–6 as fair, and scores of 7–9 as good.

## 3. Results

A total of 43 studies concerning the surgical management of scoliosis in patients diagnosed with NF1 were retrieved. These are presented in two tables. Table 1 presents articles evaluating various surgical treatment methods (either spinal fusion or growth-friendly methods) or studies on preoperative or perioperative correction techniques, including use of preoperative halo-gravity traction [11,14,15,16,17,19,20,21,22,24,25,27,28,30,31,32,33,34,35,36,37,38,40,42,44,49,50,51,52]. Among these, four are review articles and four are case series. Three studies analyse outcomes of correction on non-dystrophic cases (combined or not with dystrophic) [14,17,26]. The intraoperative guidance methods are presented in Table 2, while the use of growth-friendly systems is presented in 13 studies (Table 3); with one them focusing on the role of magnetically controlled growing rods [11,23,25,28,30,31,35,37,42,43,50].

Table 2 presents results from four studies focusing on the use of intraoperative assistance methods, including navigation and monitoring techniques [53,54,55,56]. Two of these studies are comparative analyses evaluating the use of O-arm navigation versus the free-hand technique for pedicle screw insertion [53,54]. Outcomes related with on the use of intraoperative neurophysiological monitoring are further analysed by Qiu et al., while results from surgical interventions assisted by the combined use of O-arm navigation and triggered electromyography (t-EMG) have also been reported [55,56]. 

Various postoperative complications related with the treatment of NF1 scoliosis have been reported. According to Neifert et al., the incidence of immediate postoperative complications is estimated at 2.1%, with 1.5% resulting in permanent deficits. Revision surgery was deemed necessary in 21.5% of all cases [42]. In contrast, Cai et al. reported no neurological or respiratory complications, although their study involved a smaller sample size and employed screw-based instrumentation [23,33]. Similar results were observed in patients treated with growth-friendly procedures [31]. However, several complications (mainly rod breakage) related with growth-friendly methods were presented by Carbone et al. [11]. Finally, no revision surgeries were required among sixteen patients with NF1 scoliosis who underwent single-stage posterior pedicle screw fixation, as reported by Wang et al. [21].

Correction of curvature varies among the studies retrieved. In non-dystrophic cases, curvature correction was estimated at 62.9%, whereas correction rates in dystrophic cases differed depending on curve location—thoracic versus combined thoracolumbar and lumbar curves [14]. In cases managed with growing rods until definitive fusion, the correction rate at final follow up was 50.1%, while T1-S1 (thoracic 1-sacral 1) growth was estimated at approximately 11.2 mm per year [25]. For cases treated with screw-based instrumentation, major curve angles improved from a preoperative mean of 66.1° ± 16.2° to 31.1° ± 14.6° postoperatively [32]. Similarly, Mladenov et al. reported a mean curvature correction of 54%, while annual thoracic growth appeared to be preserved [35]. 

## 4. Discussion

### 4.1. Treatment of Non-Dystrophic Scoliosis

The presence or absence of bone dystrophic features in patients with NF1 scoliotic deformities classifies the curvature as either dystrophic or non-dystrophic, respectively. Although non-dystrophic scoliosis often resembles idiopathic scoliosis, these curves may still demonstrate progression over time and, in some cases, evolve into dystrophic patterns, which usually necessitate surgical treatment. According to current literature, relatively few studies specifically address the outcomes of surgical treatment for non-dystrophic scoliosis in the NF1 population. The largest study to date was conducted by Lyu et al., who compared outcomes of single-stage, posterior-only pedicle screw instrumentation between patients with NF1-associated non-dystrophic scoliosis and those with adolescent idiopathic scoliosis. Comparable postoperative clinical outcomes and complication rates in both groups supported the efficacy of this surgical approach, even in patients with differing spinal flexibility. Nevertheless, anatomical challenges such as thin pedicles or dural ectasia in patients with non-dystrophic NF1 scoliosis may complicate pedicle screw placement [26]. 

Li et al. [17] reported three cases of non-dystrophic NF1 scoliosis treated with posterior instrumented fusion, while Halmai et al. [14] described one additional case. In Halmai’s case, a 16-year-old patient underwent posterior fusion for a non-dystrophic curve, achieving a 69.2% correction, with no pseudarthrosis [14]. No pseudarthrosis was noted and correction loss was 5° in the frontal plane and 4° in the sagittal, upon achieving final fusion. Similarly, mean postoperative coronal curve correction in non-dystrophic patients was 61.3%, with 3° correction loss in the sagittal plane and 6° in the frontal, as estimated during follow-up, respectively [17]. One of these cases involved a curve exceeding 90°, yet effective correction (60%) was achieved. This success was attributed to good preoperative bending flexibility and the use of halo vest and Cotrel gravity traction, which facilitated soft tissue relaxation. Ultimately, this patient was managed successfully with posterior-only fusion.

### 4.2. Treatment of Dystrophic Scoliosis 

#### 4.2.1. Spinal Fusion Techniques 

**Figure 3:**
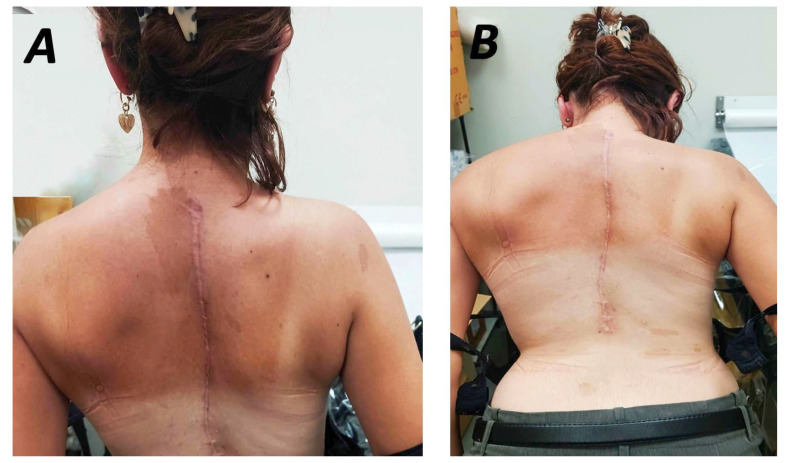
Postoperative follow-up after 3 years of a NF-1 patient with scoliosis displaying increased correction of shoulder (**A**) and trunk asymmetry (**B**) at the clinical examination.

Spinal fusion achieved with bone grafts or instrumentation (anterior and posterior approaches) was described in 24 studies [17,18,19,21,22,23,24,26,28,29,31,32,33,35,36,37,38,42,44,45,51,57]. No consensus regarding the type or time of intervention has been established. However, due to the progressive nature of NF1-associated deformities, early treatment, which involves early definitive fusion or growing rod methods, is required. Early fusion is usually performed in patients with short and sharp curves or patients over 10 years old with long curves [28]. A posterior-only approach has been suggested for less severe deformities [15]. Outcomes on the use of this approach have been reported by various studies. Wang et al. used a single-stage pedicle screw instrument system, achieving satisfactory coronal and sagittal balance, without requiring revision surgery (Figure 2D). However, global spinal balance remained an area for improvement (Figure 3A,B) [21]. Similarly, the use of multiple anchor point systems (third-generation instrumentation systems) in posterior-only fusion procedures resulted in significant correction of the mean coronal Cobb angle (58.7%) and apical vertebral rotation, with only 2.3% loss of Cobb angle correction at final follow-up [24]. These systems enhance correction stabilisation, avoiding extended correction attempts and concomitant neurological complications. The impact of implant density in the posterior surgical approach has been evaluated in relation to immediate postoperative coronal correction and loss of correction at follow-up. Both parameters presented statistically significant improvement in short-term follow-up when a higher level of implant insertion was used [54]. However, research assessing mid- and long-term outcomes of posterior-only fusion have reported that, despite good initial coronal curve correction, a higher rate of correction loss and alignment complications may occur over time. The incidence of neurological complications was lower, and when short fusion segments were selected, lung function was generally preserved [31]. 

A two-stage procedure, combining ring anterior release and fusion followed by posterior correction and fusion, was carried out in 32 patients with severe rigid deformities, who were treated with multisegmental instrumentation systems [18]. No implant failure and only two cases of pseudarthrosis were described, while correction rate and loss of correction were greater in large kyphotic curves, consistent with recent literature findings [17,18,58]. Various instrumentation systems have been used in dual-approach procedures, including hooks, pedicle screws, all-hook systems, or hybrids. Comparative studies evaluating pedicle screws versus hybrid systems revealed no statistically significant differences in coronal or sagittal correction rates or in loss of sagittal alignment. However, a statistically significant difference in coronal correction loss was observed at long-term follow-up, with less correction loss at 9.5 years postoperatively in patients treated with pedicle screw instrumentation [27]. Long-term results in a cohort of 11 patients who underwent anteroposterior fusion for dystrophic curves, with subtotal tumour resection of the concave area in seven of them, demonstrated a trend toward shorter final height compared to the general population and progression of the deformity in all cases [36]. Finally, the inclusion of neutral and stable vertebrae (in both the coronal and sagittal planes) and coronal curves exceeding 40° in long segmental posterior-only fusion yielded similar results with combined anterior and posterior approaches in patients with NF1 and scoliotic curves between 40° and 90° [17]. Both the correction rate and postoperative correction loss was comparable with results from current literature. However, the study highlights the need for dual fusion procedures in patients younger than 10 years, as this population may suffer from the crankshaft phenomenon when treated with posterior-only fusion [13]. 

#### 4.2.2. Growth-Friendly Methods

**Figure 4:**
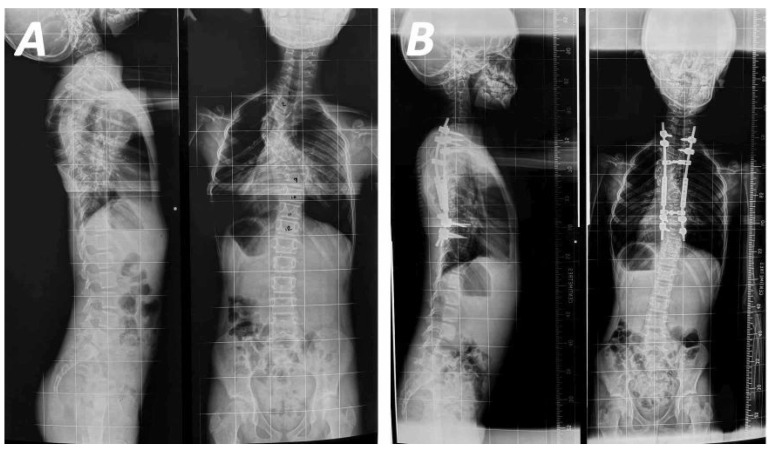
10-year-old female patient with NF1 without neurological symptoms suffering from left thoracic scoliotic deformity of 48° extending from T2 to T8 with apex at T4 vertebrae (**A**) treated with posterior instrumentation and application of screws and magnetically controlled growing rods resulting in a scoliotic curve correction to 23° (**B**).

**Figure 5:**
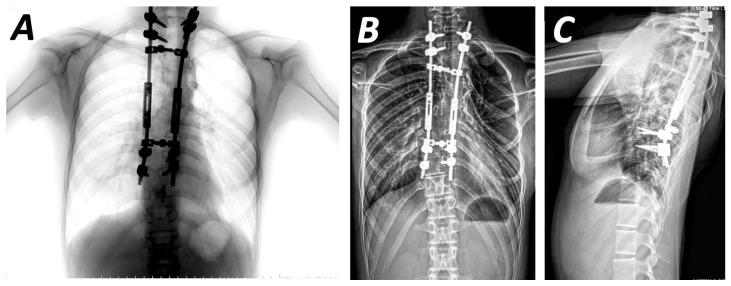
Postoperative radiographs of juvenile patient with NF1 after the lengthening of the growing rods that resulted in the correction of the deformity at the range of 16° (**A**). The 10 years of follow -up demonstrated maintenance of the correction without any clinical or imaging progression of the curve (**B**,**C**).

Clinical outcomes following use of growth-friendly methods (Figure 4A,B) have been discussed in 13 studies (Table 3) [11,23,25,28,30,33,35,37,39,42,43,47,50]. These methods have demonstrated acceptable correction with maintenance of trunk growth (Figure 5A–C). The first study, focusing exclusively on a cohort with NF1-associated scoliosis, reported similar correction rates and T1-S1 growth with other studies on the use of growing rods for early-onset scoliosis [25]. Moreover, a significant complication of this technique was proximal junctional kyphosis, attributed to low bone mineral density and dystrophic changes in patients with NF1 scoliosis, leading to poor fixation points. According to Carbone et al., Cobb angle correction improved by up to 43.4% at last follow-up, with concurrent improvement in coronal balance [11]. The best results were observed immediately after lengthening procedures, performed annually. The decrease in lengthening gain, commonly referred to as the “law of diminishing returns”, typically observed after each lengthening procedure, was not confirmed in this case, where lengthening was performed annually, in contrast to other studies. The combined use of a dual growing rod system and preoperative halo-gravity traction has been shown to facilitate the treatment of rigid deformities, achieving a Cobb angle correction of approximately 41.6% after halo-gravity traction and 53.3% at final follow-up and, without neurological disorders or autofusion [30]. Finally, only a few studies have assessed the use of magnetically controlled growing rods (MCGR) for the treatment of NF1-associated scoliosis. Although this system has demonstrated improvements in major curve correction and spinal height, its application is limited by reduced imaging visibility, particularly in patients with intraspinal tumours [47].

Tauchi et al. were the first to compare surgical results between early fusion methods (posterior-only and combined approach) versus growth-friendly methods [37]. Greater curvature correction with fewer surgical interventions was achieved in the population treated with early fusion. However, although growth-friendly methods achieved better spine and thorax growth, the final absolute height showed no statistically significant difference. Similarly, complication rates did not differ significantly. However, growth-friendly methods were associated with a higher incidence of complications and lower curve correction, as compared to fusion methods [37].

#### 4.2.3. Preoperative Planning—Three-Dimensional Printing Technology

Treatment of spinal deformities in dystrophic NF1 remains a challenge, as the complex morphology of pedicles may affect the identification of insertion points for screw placement. Therefore, defective manual insertion of the screws may result in neurological side effects and damage to vascular tissues or vital organs [59]. Furthermore, the vertebral pedicles in these patients can be narrowed, hypoplastic (concave site) or even absent, being accompanied by vertebral body rotation, which directly affects surgical outcomes, leading to pedicle root fractures and nerve root or spinal cord injuries [60]. 

**Figure 6:**
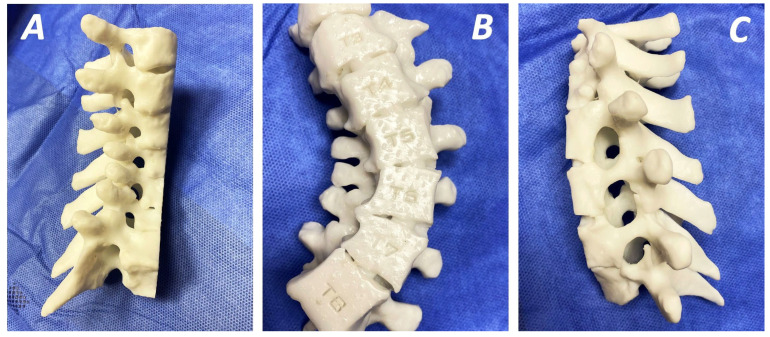
Three-dimensional printing of dystrophic scoliotic thoracic deformity of a patient with NF1 used for preoperative planning and surgical guidance to improve surgical outcomes (**A**–**C**). Note the characteristic dysplastic and narrow pedicles.

The use of three-dimensional printing (3DP) templates for navigation (Figure 6) in these types of scoliosis correction surgeries assist in optimising preoperative planning as they enhance the accuracy of screw placement and shorten surgical time and blood loss [60]. Furthermore, 3DP navigation systems optimise the surgeons’ learning curve, as they can be used for better understanding of anatomical structures, or even surgical rehearsal [61]. Especially, in cases with a Cobb angle greater than 50°, their application as accurate templating systems results in optimised intraoperative approaches, less radiation, fewer complications, and a decreased rate of revision surgeries in paediatric patients [62]. Furthermore, the combination of 3DP and pedicle guider, which is based on a drill template, can aid in precise screw placement and limited surgical time [63]. Finally, a five-point navigation template using a point-contact system has been demonstrated to reduce blood loss and prevent stripping of the posterior structures, while its segmented design helps to mitigate incompatibility issues arising from changes in body positioning [60]. 

#### 4.2.4. Intraoperative Assistant Methods 

Several intraoperative guidance systems, including O-arm navigation and intraoperative neurophysiological monitoring, have been shown to assist in more accurate pedicle screw insertion and higher implant density. According to Li et al., increased implant density leads to higher coronal correction rate (immediate) with lower correction loss and better clinical scores [54]. This implant density, reflecting safer and more accurate pedicle screw insertion in severe deformities, was achieved with the use of the O-arm. Similarly, higher implant density, better curvature correction with lower incidence of median screw perforation was feasible with the guidance of O-arm navigation, when compared to a free-hand technique [53]. Use of intraoperative neurophysiological monitoring can be safely applied even in patients with preoperative dystrophic features. However, the presence of preoperative dystrophy and neurologic deficits can lead to higher rates of failed intraoperative neurophysiological monitoring. The overall success rate of somatosensory evoked potentials and motor evoked potentials is estimated approximately 87% and 94.6%, respectively [55]. Careful assessment of preoperative neurological status assists in safer monitoring and, consequently, safer and more accurate surgical intervention. Finally, the combination of methods (t-EMG with O-arm-assisted pedicle screw placement) used in treatment of NF1 scoliosis has already been studied and appears to present 100% sensitivity, 66.7% positive predictive value, and 96.2% specificity [56].

#### 4.2.5. Post-Operative Management

Surgical interventions for the treatment of spinal deformities in patients with NF1 have already been analysed and remain the standard of care in dystrophic cases. However, it seems critical to evaluate the clinical characteristics and special needs of these patients in order to optimise their final interventional outcomes by reducing the rate of pseudarthrosis and revision surgeries. According to the literature, postoperative pseudarthrosis presents in approximately 25% of all cases and a clinically robust fusion is noted in as few as 7% of them [64]. Taking these data into account, many studies investigated the impact of several pharmacological agents on the postoperative outcomes of NF1 spinal deformities, reporting promising results. In specific, Mitogen-activated protein kinase (MEK) inhibitors presented increased activity in the reduction in spinal neurofibroma burden, or bone healing and successful spinal fusion [65]. Biphosphonates, asfotase alfa, and bone morphogenic proteins have contributed to solid spinal arthrodesis, when they are used as supplementary therapy with deformities related with NF1 in vivo [66]. Moreover, the administration of asfotase alfa and bisphosphonates in combination with surgical intervention and rhBMP-2 (recombinant human Bone Morphogenetic Protein-2) resulted in solid arthrodesis and enhanced bone healing in a patient with NF1-related dystrophic scoliosis, suggesting that enhanced bone formation, resorption, and mineralisation may be associated with better outcomes [67]. Good clinical results related with the use of off-label BMPs and bisphosphonates in clinical case series with tibial pseudarthrosis could support their use for better outcomes regarding fusion, especially in cases of such surgeries [67,68]. Although their application needs further study, measurements of bone mineral density (BMD) in cases of NF1 scoliosis may also assist in the final treatment strategy by the evaluation of serum biomarkers, vitamin D, and bone turnover when combined with biomechanical finite 3D element modelling of both the concave and the convex area of vertebral bodies [64,65]. The use of this tool can provide a future theoretical background for the improvement of surgical spinal fixation devices and the analysis of specific of scoliotic deformities.

### 4.3. Strengths and Limitations

To our knowledge, this the most recent review to focus exclusively on the operative treatment of NF1-associated scoliosis. In addition to evaluating surgical methods and instrumentation, this review includes analysis of intraoperative navigation techniques. 

However, our study has several limitations. Considering the inclusion criteria, which cover only NF1-associated scoliosis, most of the studies reviewed include relatively small sample sizes, which limits both comparability and the predictive value of the findings. Furthermore, the inclusion of only English-language publications introduces the risk of language bias.

## 5. Conclusions

Dystrophic features and a tendency for rapid progression make NF1-associated scoliosis a significant surgical challenge. No consensus on a specific treatment has been achieved so far. Although no clear genotype–phenotype correlation has been established in patients with NF1-associated scoliosis [43], additional studies regarding this may facilitate the development of personalised and widely accepted surgical management strategies for this rare disease. Further investigation into preoperative radiological examination, informed by current findings, and intraoperative guidance [40], can contribute to more accurate instrument insertion, reduce intraoperative and postoperative complications, and lead to improved clinical scores.

## Figures and Tables

**Table 1:** Literature on surgical treatment of NF1-associated scoliosis.

Author (Year)	Type of Article	Number of Patients	Purpose of Study	Subtypes of NF1	Method of Treatment	Newcastle–Ottawa Score	Key Findings
Halmai et al., 2002 [14]	Case series	12	Present results from surgical treatment of dystrophic and non-dystrophic deformities in patients with NF1	Non-dystrophic and dystrophic	In non-dystrophic posterior-only fusion. Halo-gravity traction in dystrophic and then two stages (anterior and then posterior fusion with interval of halo traction)	07 (Good)	62.9% correction of curvature in non-dystrophic cases. No dysplasia or pseudarthrosis. 4° correction loss in the frontal plane, 5° in the sagittal plane at final fusion. For dystrophic cases, mean correction loss of 5.4° in the sagittal plane and 4.8° in the frontal, at 4.4 years follow up. No pseudarthrosis and one case with neurological complications reported.
Tsirikos et al., 2004 [15]	Review	Not applicable	Review diagnosis, clinical manifestations, and treatment of spinal deformities in NF1	Non-dystrophic and dystrophic	Non-dystrophic curves treated as idiopathic scoliosis, dystrophic with aggressive treatment	-	Stabilisation of the vertebral column should be the priority rather than extensive correction, which may be accompanied by neurological impairment.
Yalcin et al., 2007 [16]	Cases series	3	Diagnosis and management of rib dislocation during deformity correction surgery in patients with NF1 dystrophic scoliosis	Dystrophic	Rib head resection in two of the cases presented, rib dislocated away from the canal during reduction manoeuvres in the other case	07 (Good)	No postoperative complications reported. Rib heads of apical convex can be dislocated in the spinal canal of these patients. Treatment with spinal instrumentation techniques (including growing rods) achieved with preventative measures.
Li et al., 2009 [17]	Research article	19	Evaluate whether extension of posterior-only fusion at one level beyond conventional one enables as efficient fusion as combined anterior–posterior approach in patients with NF1 scoliosis	Non-dystrophic and dystrophic	Posterior fusion alone in all the patients (hybrid constructs with hooks, screws and sublaminar wires)	08 (Good)	Three patients with non-dystrophic subtype. Similar results with those with combined anterior–posterior fusion (scoliosis from 40° to 90°). Correction loss greater than those treated for adolescent scoliosis but comparable with patients with NF1. One case of revision surgery presented.
Coptan and Elmiligui, 2010 [18]	Original article	32	Evaluate clinical radiological outcomes from multisegmental instrumented fusion for scoliosis with NF1	Dystrophic	Two-stage anterior release/fusion and posterior correction/fusion	08 (Good)	Correction comparable with literature results, greater corrective loss in greater kyphotic deformities.
Sun et al., 2013 [19]	Clinical article	6	Analyse results from surgical treatment of patients with NF1 deformity and intraspinal rib head insertion	Dystrophic	Posterior spinal fusion (including multilevel Smith-Petersen osteotomies) without rod head resection	07 (Good)	No neurological complications, dural tear, intracanal haematoma were reported postoperatively. Rib heads retracted away from spinal canal (5.18 × 2.6 cm). Solid spinal fusion achieved and clinical correction maintained one year after surgery.
Heflin et al., 2015 [20]	Research article	12	Evaluate results from rib-based distraction in treating patients with NF1 scoliosis and concomitant complications	Dystrophic	Vertical Expandable Prosthetic Titanium Rib implants under spinal erector fascia with rib cradles proximally inline or parallel, distally in non-ambulatory system attach pelvis with Dunne McCarthy-style pelvic S hooks. In ambulatory children, either down-going lamina hooks or pedicle screws used	08 (Good)	17 complications in 8 children. In most cases, maintenance or improvement of preop Cobb angle, progression more than 10° only 3 patients.
Wang et al., 2015 [21]	Clinical study	16	Present results from patients with dystrophic NF1 scoliosis treated with one stage posterior pedicle screw fixation	Dystrophic	Posterior one-stage pedicle screw fixation (posterior vertebral column resection in some patients). Allogeneic graft or iliac crest used	08 (Good)	No revision surgery required. One patient with transient weakness of lower extremity and one with weakness necessitating surgical exploration and intraspinal haematoma removal. Satisfactory improvement in coronal and sagittal balance. Overall, spinal balance needs to be improved. No progression of the deformity was reported.
Zhao et al., 2016 [22]	Observational study	26	Analyse radiological outcome of surgical management of dystrophic NF1-associated scoliosis	Dystrophic	Posterior column resection with intracanal rib head resection. Pedicle of hook fixation	08 (Good)	Hook dislodgement and pseudarthrosis in two patients. Extension of fusion and solid instrumentation for successful fusion results. Complete resection of intracanal rib heads (6 patients). One patient with mild transient paraparesis, postoperatively.
Cai et al., 2017 [23]	Research article	8	Evaluate results from NF1 dystrophic scoliosis with rib head protrusion in canal with posterior spinal fusion without rib head resection	Dystrophic	Posterior correction of the deformity with a pedicle screw–rod system (three-dimensional). Spinal fusion in 7 cases, one with growing rod system. No rib head resection	07 (Good)	Median spinal canal space involving intraspinal rib head was significantly lower postoperatively than preoperatively. No neurological complications at one-year follow up and maintenance of the correction.
Deng et al., 2017 [24]	Clinical article	31	Evaluate clinical efficiency of posterior-only fusion system with a multiple anchor point method	Non-dystrophic and dystrophic	Posterior-only fusion with a multiple anchor point method (as many as possible pedicle screws or hooks in key vertebrae)	09 (Good)	Significant correction at mean coronal Cobb angle and apical vertebral rotation, with only 2.3% correction loss rate of the Cobb angle. Revision required in only one case with hook dislodgement and pseudarthrosis.
Jain et al., 2017 [25]	Original article	14	Evaluate results from use of growing rods in patients with early-onset scoliosis with NF1	Dystrophic	Traditional growing rod instrumentation used (none with magnetically controlled rod)	07 (Good)	Four patients with definite fusion treatment. The correction rate was 50.1% (final follow-up), and yearly T1-S1 growth rate around 11.2 mm. 1.4 complication rate per patient (mainly proximal junctional kyphosis and proximal construct failure).
Lyu et al., 2017 [26]	Clinical study	15	Evaluate clinical outcomes from one-stage posterior fusion in patients with non-dystrophic NF1 scoliosis and compare with adolescents’ idiopathic types	Non-dystrophic	Posterior correction and posterior fusion with segmental instrumentation	07 (Good)	Similar spinal correction in both groups. Results showed the curve correction in the NF-1 group was better than patients with non-dystrophic scoliosis treated by hook–rod-based instrumentation. No apparent progression of deformity with the pedicle screw system and similar correction loss.
Wang et al., 2017 [27]	Observational study	9	Present results from management of severe rigid dystrophic scoliotic curves in NF1	Dystrophic	Combined anterior release fusion with posterior correction and instrumentation (single or two-stage procedure)	07 (Good)	No statistically significant difference in coronal and sagittal curve correction between pedicle screw and hybrid instrumentation construct. Less reduction in coronal correction in the pedicle screw group, but without statistically significant difference in loss of sagittal correction.
Yao et al., 2018 [28]	Research article	59	Evaluate incidence and risk factors for complications after surgical treatment for NF1-associated scoliosis	Dystrophic	Instrumented-based surgical treatment (pedicle screw–rod and growing rod methods)	09 (Good)	Seventeen patients with a total of nineteen complications (no neurological complications referred). Age less than nine years, kyphosis exceeding 50°, and growing rod implantation are risk factors for complications.
Carbone et al., 2019 [11]	Original article	7	Study results of growth-friendly instrumentation in management of early-onset NF1-associated scoliosis and evaluate “law of diminishing returns” effect	Dystrophic	Posterior double growing rod implantation without fusion applied in patients with dystrophic type of NF1. Lengthening performed every twelve months	07 (Good)	Three patients treated with initial instrumentation removal and final fusion. Several complications, mainly rod breakages. Comparable results to literature with the advantage of one lengthening operation annually. Law of diminishing returns not confirmed.
Yao et al., 2019 [29]	Research article	59	Evaluate outcomes and instrumentation complications in patients for NF1 dystrophic scoliosis	Dystrophic	Posterior fusion only (instrumented) or combined with anterior fusion, compared to the growing rod approach	09 (Good)	Early fusion (definitive) in patients approaching skeletal maturity or with short and sharp curves. Use of growing rod shows higher complication rate and lower corrective one. Final fusion when more instrument-related complications presented and less growth feasible after distraction.
Xu et al., 2019 [30]	Original article	11	Present results from combined use of halo-gravity traction and dual growing rod in dystrophic NF1 scoliosis	Dystrophic	Use of preoperative halo-gravity traction up to 50% of body weight and placement of 2 rods. Lengthening of 1–2 cm achieved every 6 months, until no traction feasible	08 (Good)	3.9 times of lengthening with average distance of 1.6 cm. One patient with hook dislodgement. No autofusion reported.
Cai et al., 2020 [31]	Research article	16	Compare outcomes of posterior fusion with growth-friendly treatment of dystrophic NF1 scoliosis	Dystrophic	Posterior fusion with rod derotation and in situ reduction (translational) with compression or distraction manoeuvres, growth-friendly applied with 2 rods, hooks or pedicle screws, and autologous bone and allograft at foundation area	08 (Good)	Major curve of 3 patients decreased postoperatively in growth-friendly group, while 4 patients in the other group with major curve progression. No neurological complication, no statistically significant complications between the groups. Favourable T1-S1 growth in the first group.
Cai et al., 2020 [32]	Research article	10	Compare mid–long-term results from posterior-only instrumented fusion in NF1 patients with early-onset dystrophic scoliosis	Dystrophic	All patients with screw-based instrumentation	07 (Good)	High incidence of alignment complications (only one required revision surgery). Major curve correction from 66.1° ± 16.2° preoperatively, to 31.1° ± 14.6° postoperatively. No neurological complication or lung function deterioration.
Cai et al., 2020 [33]	Research article	27	Present surgical treatment and prognosis in 27 patients with NF1-associated dystrophic deformities	Dystrophic	Various procedures related to the grade of deformity/pontodestomy or lower articular surface resection and fusion or apical vertebral body or discectomy (upper) and fusion	08 (Good)	Obvious correction in many cases, instrument (case) dislocation or rod breakage described (required revision surgery). Major curve correction without significant difference between two or three groups (certain correction of the surgical programme).
Li at al., 2020 [34]	Research article	37	Report impact of screw/hook insertion in retraction of rib head from spinal canal in patients with dystrophic NF1 scoliosis	Dystrophic	All pedicle screws constructs for 21 patients and hybrid hook screw for 16 patients.	09 (Good)	No significant different results regarding kyphosis correction and spinal height between screw/hook and non-screw/hook group. Screw/hook placement associated with higher Cobb angle and vertebral translation correction, three-dimensional relationship between spinal canal and rib head could be changed through traction and derotational withdrawal.
Mladenov et al., 2020 [35]	Annual issue article	33 (3 of them with cervical deformities)	Report outcomes from surgical treatment of skeletal deformities in patients with NF1 and spinal deformities	Dystrophic	11 patients treated with definite fusion, 11 with growth-preserving techniques, 7 with combination, 5 with preserving methods convert to fusion	08 (Good)	Good results regarding curve correction (mean 54%). Preservation of annual thoracic spine growth. Posterior approach in curves less than 60°, combination of approaches in greater curves. Use of laminar hooks or sublaminar brands in dystrophic areas.
Tauchi et al., 2020 [36]	Original article	11	Present long-term results from definitive spinal fusion for early-onset scoliosis in NF1 patients	Dystrophic	Halo traction for 2 to 3 weeks in curves larger than 80°, then anterior release bone grafting and intervertebral disc removal (via thoracotomy). Finally, posterior spinal fusion. 7 cases with subtotal tumour resection on concave side and rib strut grafting	07 (Good)	Patients shorter than general population (early definitive fusion and large scale of fusion applied). Stable instrumentation achieved (longer fusion levels). Circumferential approach, with subtotal tumour resection, rib strut grafting.
Tauchi et al., 2020 [37]	Original article	26	Compare results from early and growing rods in patients with NF1 dystrophic scoliosis	Dystrophic	Early fusion (anterior and posterior or posterior-only) versus growing rod (final fusion at mean age of 12.7 years)	08 (Good)	Greater correction of curvature in the early fusion group, with fewer surgical procedures, growing rod allows continued growth in the thorax and spine.
Li et al., 2021 [38]	Clinical article	39	Present specific features related to treatment of dystrophic NF1-associated lumbar scoliosis	Dystrophic	Posterior-only fusion approach or combined posterior–anterior/anterior–posterior	09 (Good)	No difference in pain or function scores between two groups. Anterior approach to enhance spinal fusion and stability, while reducing rod breakage and revision. Reduces growth asymmetry and prevents crankshaft phenomenon.
Marrache et al., 2021 [39]	Review article	Not applicable	Highlight natural history, management and imaging surveillance of spinal deformities in NF1 scoliosis	Dystrophic	Skeletally immature patients with non-dystrophic NF1 scoliosis treated with brace, with 20° to 40° curves. Patients with curves under 20°, only observation follow-up every six months, curves over 45° with early fusion or growth-friendly instrumentation. Patients with dystrophic curves under 20° clinical observation every six months, surgically treated upon progression (relative indication).	-	Annual clinical and scoliosis examination for children aged 1 to 5 years with NF1. Growing rods compared to early fusion allow spinal lengthening, but early fusion can result in similar correction with fewer procedures. Magnetically controlled growing rods contribute to growth lengthening by an external magnet, avoiding additional surgical procedures.
Pushpa et al., 2021 [40]	Case series	10	Present a radiological evaluation of morphological alterations of ten dystrophic scoliotic curves and impact on surgical management	Dystrophic	Not applicable	07 (Good)	A wide spectrum of anatomical changes, with gross variations even with small curves, 34% of pedicles in apex and three adjacent segments (above and below) safe for instrument insertion. Need for CT-guided preoperative planning.
Mao et al., 2022 [41]	Case series	15	Present risk factors for convex coronal imbalance and improve manoeuvres for postoperative coronal balance	Dystrophic	4 patients with combined staged treatment, 11 patients with posterior-only fusion	07 (Good)	For thoracolumbar/lumbar convex coronal imbalance limited and unreliable distal screw purchases with poor correction of lumbosacral curve, leave residual take of angle, risk of failure of coronal rebalance.
Neifert et al., 2022 [42]	Systematic review	30 studies (761 patients)	Present natural history, treatment options and outcomes in patients with dystrophic scoliosis related to NF1	Dystrophic	Different treatment: posterior-only fusion, anterior and posterior approach, growth-friendly methods (hybrid constructs, pedicle screw-only constructs, and hook-based constructs)	-	Immediate postoperative neurological complication was 2.1%, rate of permanent neurological deficits was 1.2%. Revision surgery rate was 21.5%.
Li et al., 2022 [43]	Research article	14	Analyse genotype, outcomes in patients with NF1-associated dystrophic scoliosis	Dystrophic	Growing rods (lengthening at 6–12 months) or posterior spinal fusion	07 (Good)	Twelve patients with pathogenic variants. No clear association between genotype and phenotype, no mutation hotspot on NF1 gene.
Price et al., 2022 [44]	Original article	533	Report outcomes from multilevel fusion surgery	Dystrophic	Multilevel spinal fusion	09 (Good)	NF1 patients with higher risk of neurological impairment. No statistically significant difference in mortality (in-hospital), resource utilisation and quality-based outcome.
Wu et al., 2022 [45]	Original article	46	Compare results of a hybrid method of segmented correction (lacking pedicle screws in apical area) with these of the traditional in patients with dystrophic NF1 scoliosis	Dystrophic	Halo-gravity traction preoperatively in both groups. After pedicle screw placement and second level osteotomy of the apical area performed, two rods placed on concave side connected with tulip connectors and locked after distraction	08 (Good)	No statistically significant differences regarding spinal flexibility, level of fusion, blood loss, age at operation or time of operation. Higher average Cobb angle correction with lower postoperative loss of correction in the group with segmented correction. No pseudarthrosis or fixation failure reported in either group.
Zhao et al., 2022 [46]	Original article	53	Compare radiographical and clinical results from a 2 rod and sectional correction technique in patients with dystrophic NF1 scoliosis	Dystrophic	Sectional procedure included two short rods at concave side of curve connected with domino, compression manoeuvre of rods to correct concave curve, installation of long rod at the convex. Traditionally performed with two rods (known method)	09 (Good)	Better results regarding coronal balance distance in sectional group, with less correction loss postoperatively. No implant failure reported, due to prevention of pedicle screw removal throughout translation, rod rotation or screw loosening as a result of excessive stress.
Xu et al., 2023 [47]	Original article	Not applicable	Emphasise the role of magnetically controlled growing rods (MCGRs) and definitive spinal fusion on treatment of children with NF1.	Dystrophic	Recommendations on thoracolumbar and cervical deformity correction (four contraindications for use of magnetically controlled growing rods)	-	Use of magnetically controlled growing rods associated with improvements in curve magnitude and spinal height. Diminished visualisation when imaging the postoperative spine.
Dastagirzanda et al., 2024 [48]	Research article (narrative review)	2	Present two cases of NF1 dystrophic scoliosis with vertebral subluxation	Dystrophic	Use of halo-gravity traction in the first case without change in subluxation and then fusion. In the second case, neck haematoma and respiratory failure, necessitating embolization while patient was on halo-gravity traction. The same patient, while on rehabilitation, led to paraplegia, as a result of a fall, which was treated with four-rod construct accompanied with C7 to T2 laminectomy and adequate spinal cord decompression	07 (Good)	Unpredictable progression and complication of curves in both cases (following skeletal maturity). Rare complications of halo-gravity traction, which can be unsuccessful as in the first case or cause artery injuries. Preoperative planning of deformity correction should include plexiform tumour resection, tissue defects, and dural ectasia.
Liang et al., 2024 [49]	Original article	15	Evaluate safety, effectiveness of use of halo-gravity traction and growing rod system in the treatment of patients with early-onset NF1-associated scoliosis	Dystrophic	Traction duration for more than 12 h. Traditional growing rods applied with lengthening interval of 9–12 months	07 (Good)	No complications associated with halo-gravity traction, one-rod dislocation and one breakage of rod reported. Correction rate of Cobb angle with growing rod surgery was 47.67%. Better results regarding trunk balance, correction rate, and spine height.
Wang et al., 2024 [50]	Review article	37 articles (1032 patients)	Review current literature on natural history, clinical features and surgical managements of NF1 spinal deformities	Dystrophic	24 studies with bone-grafted or instrument-based spinal fusion, growing rod used in two of them. 4 comparative studies of both treatment methods	-	Satisfactory results for Cobb angle, sagittal kyphosis, and the T1-S1 length of the spinal in patients treated with spinal fusion. Acceptable results from the use of growing rods, but showed lower curve correction, higher incidence of complications not capable of correcting the sagittal kyphosis. Small population of study on this treatment method.
Lo et al., 2025 [51]	Research article	126	Compare outcomes of patients with NF1 dystrophic scoliosis after three column osteotomies, halo-gravity traction, and posterior column osteotomy	Dystrophic	Posterior column osteotomy performed in all three patient groups	09 (Good)	Statistically significant postoperative results observed in main curve Cobb angle, apical vertebral translation, segmental kyphosis, and deformity angular ratio in all three groups. No significant loss of correction reported, and coronal imbalance showed significant improvement in both the halo-gravity traction and posterior column osteotomy group.

**Table 2:** Literature on intraoperative guidance methods.

Author (Year)	Type of Article	Number of Patients	Purpose of Study	Subtypes of NF1	Method of Treatment	Newcastle- Ottawa Score	Key Findings
Jin et al., 2015 [53]	Original article	32	Compare accuracy of insertion pedicle screw with O-arm navigation with free-hand technique in dystrophic NF1-associated scoliosis	Dystrophic	Pedicle screw posterior procedures	07 (Good)	Statistically greater correction of the main curve in the O-arm group. Higher accuracy in screw position in the O-arm group. Lower incidence of medial screw perforation and increased implant density in the apical region in this group.
Li et al., 2017 [54]	Clinical article	41	Evaluate implant density, radiological, clinical outcomes in NF1 dystrophic thoracic scoliosis	Dystrophic	Posterior pedicle screw or hybrid instrumentation with hook (free-hand or O-arm navigation technique)	08 (Good)	Better immediate postoperative coronal correction rate with implant density higher than 1.35, less loss of correction at follow-up.
Qiu et al., 2021 [55]	Clinical article	92	Evaluate data from intraoperative neurophysiological monitoring during management of NF1 dystrophic scoliosis and risks from failure	Dystrophic	Posterior spinal deformity correction and fusion	09 (Good)	17 patients with failed intraoperative neurophysiological monitoring, associated with more dystrophic features and preoperative neurological deficits. The overall success rates of SEP (somatosensory evoked potentials) and MEP (motor evoked potentials) were 87.0 and 94.6%.
Shao et al., 2021 [56]	Research article	65	Report results from triggered electromyography (t-EMG) with O-arm-assisted pedicle screw placement in treatment of NF1 scoliosis	Dystrophic	Posterior thoracolumbar spinal fusion with (T1-S1) with t-EMG and O-arm-assisted screw placement	08 (Good)	3 malpositioned screws (2 patients) were not detected with O-arm, but only with t-EMG (all of them in the periapical area). Combination of methods associated with 100% sensitivity, positive predictive value of 66.7% and specificity of 96.2%.

**Table 3:** Literature on growth-friendly methods.

Author (Year)	Type of Article	Number of Patients	Purpose of Study	Method of Treatment	Key Findings
Cai et al., 2017 [23]	Research article	8	Evaluate results from patients with NF1 dystrophic scoliosis with rib head protrusion in spinal canal treated posterior spinal fusion without rib head resection	Posterior correction of deformity with pedicle screw–rod system (three-dimensional). Spinal fusion performed in 7 cases, one with growing rod system. No procedure involved rib head resection	Median spinal canal space involving intraspinal rib head significantly lower postoperatively than preoperatively. No neurological complications at 1-year follow-up correction maintenance
Jain et al., 2017 [25]	Original article	14	Evaluate results from the use of growing rods in patients with early-onset NF1-associated scoliosis	Traditional growing rod instrumentation used (none with magnetically controlled rod)	Four patients with definite fusion. Correction rate 50.1% (final follow-up), yearly T1-S1 growth rate 11.2 mm. 1.4 complication rate per patient (mainly proximal junctional kyphosis and proximal construct failure)
Yao et al., 2018 [28]	Research article	59	Evaluate incidence and risk factors for complications after surgical treatment for NF1-associated scoliosis	Instrument-based surgical treatment (pedicle screw–rod and growing rod methods)	17 patients with total of 19 complications (no neurological). Age under 9 years, kyphosis > 50°
Carbone et al., 2019 [11]	Original article	7	Study results of growth-friendly instrumentation in the management of early-onset NF1-associated scoliosis and evaluate the “law of diminishing returns” effect	Posterior double growing rod implantation without fusion applied in patients with dystrophic NF1. Lengthening performed every twelve months	3 patients with initial instrumentation, removal and final fusion. Rod breakages. Advantage of 1 lengthening operation annually
Xu et al., 2019 [30]	Original article	11	Present results from combined use of halo-gravity traction and a dual growing rod method in dystrophic NF1 scoliosis	Use of preoperative halo traction up to 50% of body weight and surgical placement of 2 rods. Lengthening of 1–2 cm achieved every 6 months, until no traction feasible	3.9 times of lengthening with average distance of 1.6 cm. One patient with hook dislodgement. No autofusion reported
Cai et al., 2020 [31]	Research article	16	Compare outcomes of posterior fusion and with growth-friendly treatment of dystrophic NF1 scoliosis	Posterior fusion with rod derotation and in situ reduction (translational) with compression or distraction manoeuvres, growth-friendly applied with 2 rods, hooks or pedicle screws and autologous bone and allograft at the foundation area	Major curve of 3 patients decreased postoperatively in growth-friendly group, while 4 patients in the other group with major curve progression. No neurological complications, no statistically significant complications between two groups. Favourable T1-S1 growth in the first group
Mladenov et al., 2020 [35]	Annual issue article	33 (3 of them with cervical deformities	Report outcomes from surgical treatment of skeletal deformities in paediatric patients with NF1 and spinal deformities	11 patients treated with definite fusion, 11 with growth preserving techniques, 7 with a combination of both, and 5 treated with preserving methods convert to fusion	Good results regarding curve correction (mean 54%). Preservation of annual thoracic spine growth. Posterior approach in curves under 60°, combination of approaches in greater curves. Use of laminar hooks or sublaminar brands in dystrophic areas
Tauchi, et al., 2020 [37]	Original article	26	Compare results from early and growing rods in patients with NF1 dystrophic scoliosis	Early fusion (anterior and posterior or posterior-only) versus growing rod (final fusion at mean age of 12.7 years)	Greater correction of curvature in the early fusion group, with fewer surgical procedures, growing rod allows continued growth in the thorax and spine
Marrache et al., 2021 [39]	Review article	Not applicable	Highlight natural history, management and imaging surveillance of spinal deformities in NF1 scoliosis	Skeletally immature patients with non-dystrophic NF1 scoliosis treated with brace when curves 20° to 40°. Curves under 20° only observation every six months, exceeding 45° early fusion or growth-friendly instrumentation. Dystrophic curves under 20° observation every 6 months, treated when curvature progressing	Annual examination for children aged 1 to 5 years. Growing rods compared to early fusion spinal lengthening can result in similar correction. Magnetically controlled growing rods contribute to growth lengthening, avoiding surgical procedures
Neifert et al., 2022 [42]	Systematic review	30 studies (761 patients)	Present natural history, treatment options and outcomes in patients with NF1-associated dystrophic scoliosis	Different treatment options: posterior-only fusion, anterior and posterior approach, growth-friendly methods	Immediate postoperative neurological complication was 2.1%, rate of permanent neurological deficits 1.2%. Revision rate 21.5%
Li et al., 2022 [43]	Research article	14	Analyse genotype and surgery outcomes in NF1-associated dystrophic scoliosis	Growing rods (lengthening at 6–12 months) or posterior spinal fusion	12 patients with pathogenic variants. No clear association genotype and phenotype, no mutation hotspot on NF1 gene
Xu et al., 2023 [47]	Original article	Not applicable	Emphasise the role of magnetically controlled growing rods (MCGRs) and definitive spinal fusion in the treatment of children with NF1	Recommendations on thoracolumbar and cervical deformity correction (four contraindications for the use of magnetically controlled growing rods)	Use of magnetically controlled growing rods with improvements in curve magnitude and spinal height. Diminished visualisation imaging postoperative spine
Wang et al., 2024 [50]	Review article	37 articles (1032 patients)	Review current literature on the natural history, clinical features and surgical managements of NF1 spinal deformities	24 studies with bone-grafted or instrument-based spinal fusion, growing rod used in two of them. 4 comparative studies of both treatment methods	Satisfactory results of Cobb angle, sagittal kyphosis, and T1-S1 length in spinal fusion. Acceptable results from growing rods, lower curve correction, higher incidence of complications, not capable of correcting sagittal kyphosis

## Data Availability

The data presented here are available upon request from the corresponding author.

## Supplementary Materials

The following supporting information can be downloaded at: https://www.mdpi.com/article/10.3390/jcm15010104/s1, Table S1: PRISMA 2020 checklist. Reference [69] has been cited in the Supplementary Materials.

## Abbreviations

NF1: Neurofibromatosis type 1; NF2: Neurofibromatosis type 2; cAMP: cyclic adenosine monophosphate; PI3K: phosphatidylinositol-3-kinase; MRI: Magnetic Resonance Imaging; CT: Computed Tomography; PRISMA: Preferred Reporting Items for Systematic Reviews Meta-Analyses; PROSPERO: Prospective Register of Systematic Reviews; t-EMG: triggered electromyography; T1-S1: thoracic 1-sacral 1; MCGR: magnetically controlled growing rods; 3DP: three-dimensional printing; MEK: Mitogen-activated protein kinase; rhBMP-2: recombinant human Bone Morphogenetic Protein-2; BMD: bone mineral density.
